# Insights into absence of lymphoma despite fulminant Epstein-Barr virus infection in patients with XIAP deficiency

**DOI:** 10.1172/jci.insight.193787

**Published:** 2025-07-15

**Authors:** Yizhe Sun, Janet Chou, Kevin D. Dong, Steven P. Gygi, Benjamin E. Gewurz

**Affiliations:** 1 Division of Infectious Diseases, Department of Medicine, Brigham and Women’s Hospital, Boston, Massachusetts, USA.; 2 Center for Integrated Solutions for Infectious Diseases, Broad Institute, Cambridge, Massachusetts, USA.; 3 Division of Immunology, Department of Pediatrics, Harvard Medical School, Boston Children’s Hospital, Boston, Massachusetts, USA.; 4 Department of Cell Biology, Harvard Medical School, Boston, Massachusetts, USA.

**Keywords:** Infectious disease, Virology, Apoptosis, Lymphomas

## Abstract

X-linked Lymphoproliferative Syndromes (XLP), arising from mutations in *SH2D1A* or *XIAP* genes, are characterized by fulminant Epstein-Barr virus (EBV) infection. Lymphomas occur frequently in XLP-1 and in other congenital conditions with heightened EBV susceptibility, but not in XLP-2. Why XLP-2 patients are apparently protected from EBV-driven lymphomagenesis remains a key open question. To gain insights, newly EBV-infected versus receptor-stimulated primary B cells from XLP-2 patients or with *XIAP* CRISPR editing were compared with healthy controls. *XIAP* perturbation impeded outgrowth of newly EBV-infected B cells, but not of CD40 ligand and interleukin-21–stimulated B cells. XLP-2–deficient B cells showed significantly lower EBV transformation efficiency than cells from healthy controls. Interestingly, EBV-immortalized lymphoblastoid cell proliferation was not impaired by *XIAP* knockout, implicating a XIAP role in early EBV B cell transformation. Mechanistically, nascent EBV infection activated p53-mediated apoptosis signaling, which was counteracted by XIAP in control cells. With XIAP deficiency, EBV markedly elevated apoptosis rates over the first 2 weeks of infection. IFN-γ, whose levels are increased with severe XLP2 EBV infection, markedly increased newly EBV-infected B cell apoptosis. These findings underscored XIAP’s crucial role in support of the earliest stages of EBV-mediated B cell immortalization and provide insights into the curious absence of EBV^+^ lymphoma in patients with XLP-2.

## Abstract 2

XIAP mutation impedes EBV-driven B-cell transformation and lymphomagenesis by potentiating p53-driven caspase activation and apoptosis.

## Graphical abstract

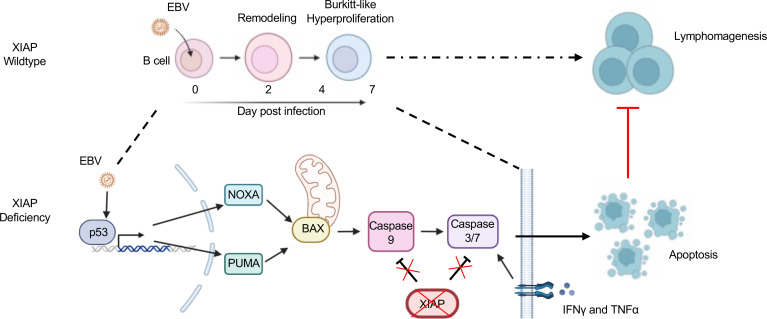

## Introduction

EBV persistently infects more than 90% of adults and contributes to over 200,000 cancers per year (1–3). While acute EBV infection is controlled by immunocompetent hosts and typically results in subclinical disease, EBV is a major pathogen in hosts with primary or acquired immunodeficiency, in particular lymphoproliferative disease (4–8). EBV is associated with multiple kinds of cancers, including lymphomas, gastric, and nasopharyngeal carcinoma (9). Defects in anti-EBV immunity increase rates of multiple EBV-associated lymphomas, including Burkitt, Hodgkin, and immunoblastic lymphomas (5, 7, 10).

EBV uses a series of latency oncogenes to drive proliferation of quiescent B cells. Over the first several days after infection, EBV uses the prelatency program, comprised of Epstein-Barr nuclear antigens (EBNA) 2, LP, and BHRF1 to activate and remodel newly infected B cells. Over days 4 to 7 after infection, cells switch to the viral latency IIb program, comprised of EBNA1, 2A, LP, 3A-C, BHRF1, and noncoding RNAs (11). Latency IIb–driven cells enter a hyperproliferative phase with markedly elevated MYC expression levels reminiscent of Burkitt lymphoma (12, 13), which results in activation of the DNA damage response (12). At approximately 1 week after infection, infected B cells transition to lymphoblastoid physiology and begin to express the EBV latency III program, comprised of 6 EBNA, 2 latent membrane proteins (LMP), and noncoding RNAs (14). LMP1 mimics signaling by CD40, a key B cell coreceptor activated by T cell CD40-ligand (CD40L) (15–17). Latency III highly upregulates BCL2 family antiapoptotic proteins (18, 19). LMP2A mimics aspects of B cell receptor signaling (20). If left unchecked, EBV transforms primary human B cells into immortalized, continuously proliferating lymphoblastoid cell lines (LCLs), which serve as a major model for EBV^+^ immunoblastic lymphomas of immunosuppressed hosts.

EBV immune control is primarily mediated by T cells. Natural killer (NK) cells and humoral responses also play crucial roles in regulating EBV activity (21). The importance of these immune mechanisms is underscored by the severe outcomes observed in individuals with compromised immunity, such as those with X-linked lymphoproliferative (XLP) syndromes (21). In these cases, defects in immune signaling pathways result in fulminant EBV infection, highlighting the critical balance between EBV and host immune responses.

Two congenital XLP syndromes have been described, which share extreme susceptibility to EBV, resulting in fulminant infectious mononucleosis, dysgammaglobulinemia, and hemophagocytic lymphohistiocytosis (HLH). HLH is a T cell and macrophage hyperactivation state resulting in histiocytic bone marrow infiltration and hemophagocytosis (22–24). XLP-1 is caused by loss-of-function mutations in *SH2D1A*, which encodes the 128 amino acid SH2-domain containing signaling lymphocyte activation molecule–associated protein (SLAM-associated protein [SAP]) (MIM no. 308240). SAP controls signaling downstream of SLAM family receptors, including CD150, CD229, 2B4, CD84, and NTB-A (25, 26). XLP-2 instead arises from congenital mutations of the X-linked inhibitor of apoptosis (*XIAP*, also termed *BIRC4*; MIM no. 300635), a 497-amino acid member of the inhibitor of apoptosis protein (IAP) family that serves as a central regulator of apoptotic cell death by inhibiting caspases 3 and 7 (25–29). XIAP has additional roles in many other pathways (30). Patients with XLP-1 and 2 each exhibit defective T and NK cell responses, including the absence of natural killer T cells (7). XLP syndromes are characterized by severe infectious mononucleosis, HLH, and a range of hematological dyscrasias (31, 32). However, while rates of HLH and splenomegaly are higher with XLP-2, there are no reported cases of EBV-associated lymphoproliferative disease in this syndrome. This stands in contradistinction to essentially all other primary immunodeficiency syndromes that manifest by susceptibility to EBV (31, 33).

A notable difference between SAP and XIAP is their cell tropism. While SAP is expressed primarily in NK, NKT, and T cells, XIAP is ubiquitously expressed (31). This disparity suggests that the lack of B cell malignancies in XLP-2 may be attributed to intrinsic factors within EBV-infected B cells, rather than from defective cell-mediated immunity. Here, we tested the role of XIAP in early versus late stages of EBV-driven B cell immortalization. *XIAP* perturbation significantly diminished proliferation of newly EBV-infected human B cells, but not of identical B cells stimulated by CD40-ligand (CD40L) and IL-4, suggesting an EBV-specific role. We present evidence that EBV subverts XIAP to counteract a p53- and BAX-dependent apoptosis pathway, otherwise triggered by nascent EBV infection. This effect was strongly exacerbated by interferon gamma, whose levels are markedly elevated in XLP-2 patients with severe EBV infection.

## Results

### EBV subverts XIAP to support newly infected primary human B cell outgrowth.

**Figure 1:**
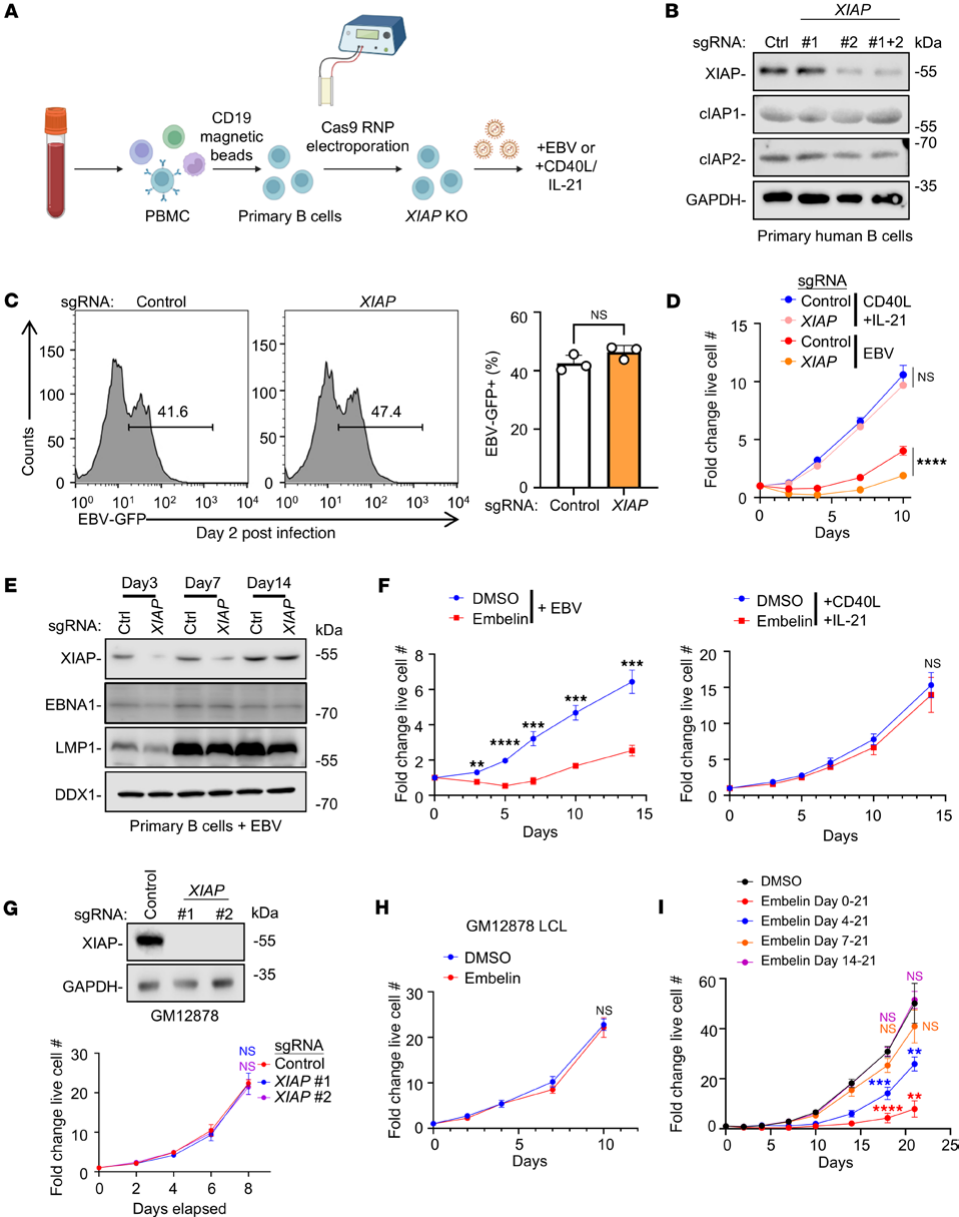
XIAP inactivation impairs the outgrowth of newly EBV-infected primary B cells. (**A**) Workflow: B cells were electroporated with Cas9 ribonucleoprotein (RNP) complexes containing *XIAP* targeting or nontargeting control single guide RNA (sgRNA). 1 hour after electroporation, cells were infected with EBV or stimulated by CD40L/IL-21. (**B**) Immunoblot analysis of whole cell lysates (WCL) from primary B cells on Day 3 after electroporation with Cas9 control or *XIAP* sgRNA-containing RNPs. (**C**) FACS analysis of control versus *XIAP* edited B cells at Day 2 after infection by EBV that expressed a GFP marker. Mean + SD GFP^+^ cell percentages from *n* = 3 replicates are shown. (**D**) Growth curve analysis of primary B cells electroporated with Cas9 RNPs and treated with CD40L/IL-21 or infected with EBV. (**E**) Immunoblot analysis of WCL from primary B cells transfected with RNP and on the indicated days after EBV infection. (**F**) Growth curve analysis of EBV^+^ (left) or CD40L/IL-21 treated (right) primary B cells treated with DMSO or the XIAP inhibitor embelin. (**G**) Immunoblot and growth curve analysis of Cas9^+^ GM12878 LCLs expressing control or *XIAP*-targeting sgRNAs. (**H**) Growth curve analysis of DMSO or embelin-treated GM12878. (**I**) Growth curve analysis of primary B cells infected by EBV at Day 0 and treated with embelin as indicated. Statistical significance was assessed by comparing each indicated groups with DMSO-treated control groups. Mean ± SD fold change cell numbers from *n* = 3 biological replicates, relative to Day 0 values, are shown (**D** and **F**–**I**). Blots are representative of *n* = 3 replicates. Blots of the same samples were run in parallel at the same time under identical conditions (**B** and **E**). Embelin (5 μM), CD40L (50 ng/mL) and IL-21 (50ng/mL) were replenished every 3 days (**D**, **F**, **H**, and **I**). Statistical significance was assessed by 2-tailed unpaired Student’s *t* test (**C**, **D**, **F**, **H**, and **I**) or 1-way ANOVA followed by Tukey’s multiple comparisons test (**G**). **P < 0.01, ***P < 0.001, ****P < 0.0001.

To characterize how XIAP deficiency impacts EBV-mediated B cell transformation, we utilized CRISPR-Cas9 editing to functionally knockout (KO) *XIAP* in primary human B cells isolated from healthy donors (Figure 1A). FACS analysis indicated that Cas9/single guide RNA (sgRNA) ribonucleoprotein complexes (RNP) were successfully delivered to over 50% of B cells (Supplemental Figure 1A; supplemental material available online with this article; https://doi.org/10.1172/jci.insight.193787DS1), and immunoblot analysis confirmed depletion of XIAP expression across the bulk population (Figure 1B).

Control versus XIAP-depleted B cells were infected with EBV, or, for cross-comparison, stimulated by CD40L and IL-21, a combination that efficiently drives B cell proliferation (34). *XIAP* editing did not significantly alter EBV infection efficiency, as judged by an EBV genomic GFP reporter that can be used to mark infected B cells (Figure 1C). However, growth curve analysis highlighted that *XIAP* editing markedly reduced the efficiency of EBV-driven primary B cell outgrowth. Intriguingly, *XIAP* KO did not significantly alter proliferation of CD40L/IL-21 treated cells, suggesting an EBV-specific, B cell intrinsic phenotype (Figure 1D). Of note, CRISPR editing depleted XIAP only in a subset of primary B cells, likely due to the inability to deliver Cas9 RNPs across the population. Immunoblot analysis highlighted that there was a selection against XIAP deficiency by day 14 after infection, suggesting that XIAP plays important roles in guiding the outgrowth of newly EBV-infected B cells (Figure 1E).

To further characterize how XIAP supports early EBV-mediated B cell outgrowth, we treated newly EBV infected versus CD40L/IL-21–stimulated primary B cells with the small-molecule XIAP antagonist embelin (35), which interacts with the same XIAP BIR3 domain residues as caspase-9 (27). Consistent with the *XIAP*-KO phenotype, embelin significantly impeded EBV-driven, but not CD40L/IL-21–induced B cell outgrowth (Figure 1F). However, consistent with prior reports (36), embelin or CRISPR *XIAP* KO did not significantly alter proliferation of 2 immortalized LCLs, GM12878 and GM15892 (Figure 1, G and H, and Supplemental Figure 1, B and C). Taken together, these results suggest that XIAP plays an important role at an early stage of EBV-mediated B cell transformation, but is dispensable following viral B cell immortalization. In support of this hypothesis, early administration of embelin impaired EBV-driven outgrowth, but its impact was nonsignificant when started at 7 days after EBV infection or at later timepoints of infection (Figure 1I). XIAP therefore plays a critical role in support of EBV-infected B cell transformation within the first week of infection.

### Newly EBV-infected but not CD40L/IL-21–driven proliferation is impaired in XLP-2 B cells.

**Figure 2:**
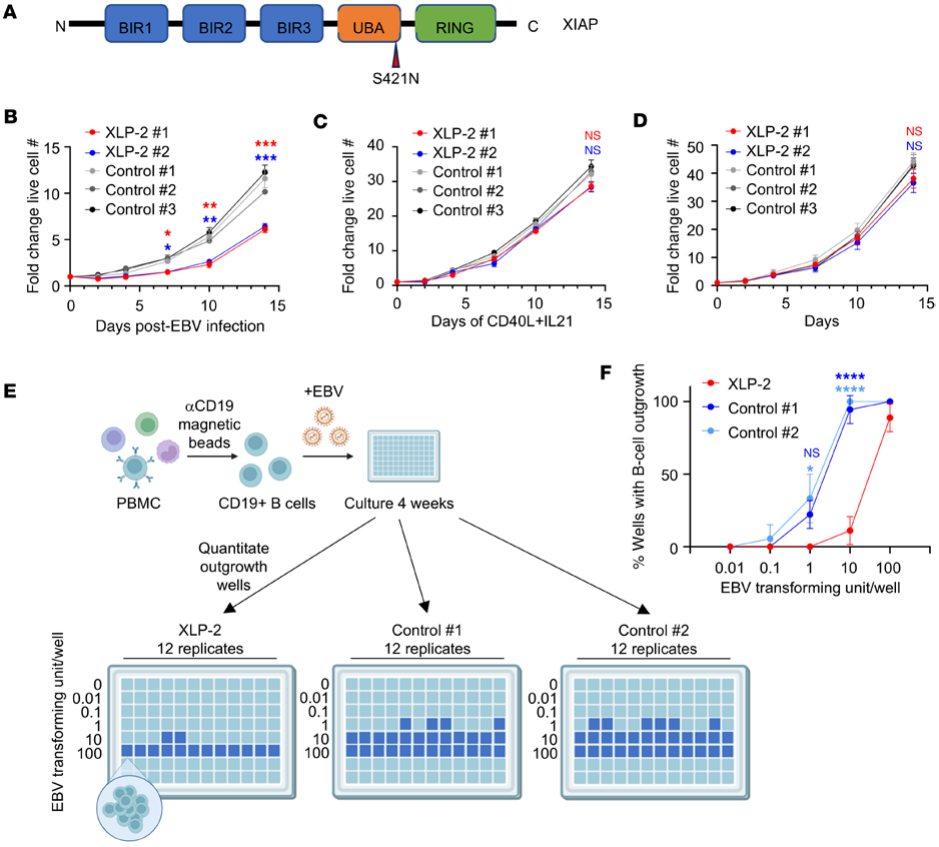
B cells from patients with XLP-2 demonstrate impaired EBV, but not CD40L/IL-21–driven outgrowth at early timepoints. (**A**) Schematic diagram highlighting the *XIAP* mutation shared by XLP-2 Patient numbers 1 and 2. (**B**) Growth curve analysis of primary B cells from patients with XLP-2 or people in the control group that were infected with EBV on Day 0. Statistical significance of comparisons between the XLP-2 samples and Control no. 1 are indicated. (**C**) Growth curve analysis of primary B cells from patients with XLP-2 or controls treated with CD40L and IL-21, which was replenished every 3 days. Statistical significance of comparisons between the XLP-2 samples and Control no. 1 are indicated. (**D**) Growth curves of lymphoblastoid cells established from B cells from Patients 1 or 2 with XLP-2, or from 3 healthy controls. Statistical significance of comparisons between the XLP-2 samples and Control no. 1 are indicated. (**E**) EBV B cell transformation assay workflow. CD19^+^ B cells purified from PBMCs were plated and infected with serial dilutions of the Akata EBV strain, using a range of 0–100 EBV transforming units/well. Wells with B cell outgrowth were scored 4 weeks later. (**F**) EBV transformation assays of primary human B cells from XLP-2 Patient no. 1 or from 2 healthy controls, as in **E**. Shown are the mean ± SD percentages of wells with B cell outgrowth from *n* = 3 replicates. Mean ± SD fold change live cell numbers from *n* = 3 replicates, relative to Day 0 values, are shown (**B**–**D**). Statistical significance was assessed by 1-way ANOVA followed by Tukey’s multiple comparisons test (**B**–**D**, and **F**). *P < 0.05, **P < 0.01, ***P < 0.001, ****P < 0.0001.

We next characterized effects of XIAP deficiency on early EBV-mediated B cell outgrowth in primary B cells from patients with XLP-2 versus from individuals in a healthy control group. Patients 1 and 2 with XLP-2, who are brothers, possess an XIAP missense variant (NP_001158.2: p.Ser421Asn) that compromises XIAP function (Figure 2A) (37). Venous blood was collected from 3 healthy donors on the same day (Controls 1–3). B cells were purified by negative selection and infected with EBV or stimulated by CD40L/IL-21. Similar to our *XIAP* CRISPR analyses, proliferation of XLP-2 cells was diminished over the first week of infection relative to healthy controls (Figure 2B). By contrast, XLP-2 and control B cells proliferated similarly in response to CD40L and IL21 treatment (Figure 2C). We observed similar effects with B cells from two additional patients with XLP-2 (Patients 3 and 4) who possess an XIAP nonsense variant (NP_001158.2: p.Arg49*) (Supplemental Figure 2, A–C). Consistent with a key early, but not late, XIAP role in support of EBV-mediated transformation, LCLs established from both patients with XLP-2 and from controls proliferated at similar rates (Figure 2D).

To further characterize *XLP-2* mutation effects on nascent EBV infection, we conducted transformation assays, in which serial dilutions of EBV are added to primary B cells, and the percentage of wells with cellular outgrowth are scored at 4 weeks after infection (Figure 2E). Consistent with our growth curve phenotypes, *XIAP* mutation significantly reduced EBV B cell transformation efficiency (Figure 2F).

### XIAP plays key antiapoptosis roles in newly EBV-infected B cells.

**Figure 3:**
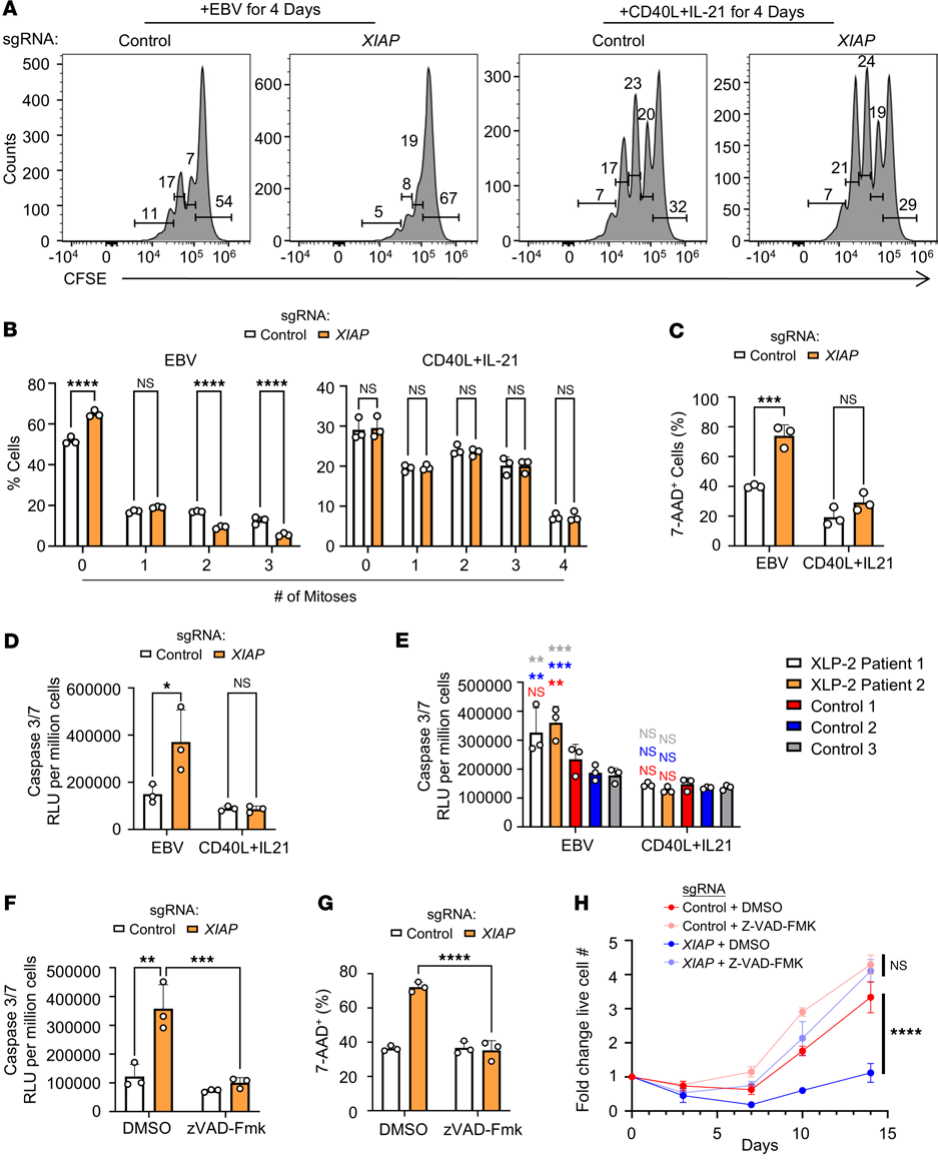
EBV, but not CD40L/IL-21, triggers apoptosis within the first week of XLP-2 B cell infection. (**A**) FACS analysis of control versus XIAP-edited primary B cells at Day 4 after EBV infection or CD40L/IL-21 stimulation. Shown are representative FACS plots from *n* = 3 replicates of cells stained with CFSE on Day 0. Live cells were gated by absence of 7-AAD vital dye uptake. (**B**) Mean + SD percentages of cells with the indicated number of mitoses from *n* = 3 replicates of EBV infection versus CD40L/IL-21 stimulation, as in **A**. (**C**) Mean + SD % 7AAD^+^ cells from *n* = 3 replicates of control or XIAP-edited primary B cells on Day 4 after EBV infection or CD40L/IL21 treatment. (**D**) Mean + SD caspase 3/7 activity from *n* = 3 replicates of control or XIAP-edited primary B cells on day 4 after EBV infection or CD40L/IL-21 treatment. (**E**) Mean + SD caspase 3/7 activity from *n* = 3 replicates of control or XLP-2 primary B cells on day 4 after EBV infection or CD40L/IL-21 treatment. (**F**) Mean + SD caspase 3/7 activity from *n* = 3 replicates of control or XIAP edited primary B cells incubated with DMSO or the pan-caspase inhibitor zVAD-fmk (20 μM) on day 4 after EBV infection or CD40L/IL-21 treatment. (**G**) Mean + SD % 7AAD^+^ cells from *n* = 3 replicates of control or XIAP-edited primary B cells on Day 4 after EBV infection or CD40L/IL21 treatment. (**H**) Growth curve analysis of control versus *XIAP*-edited B cells infected with EBV on Day 0 and cultured with DMSO or zVAD-Fmk (20 μM). Mean ± SD fold-change live cell numbers, relative to uninfected values, are shown. Statistical significance was assessed by 2-tailed unpaired Student’s *t* test (**H**) or 1- (**E**) or 2-way (**B**–**D**, **F**, and **G**) ANOVA followed by Tukey’s multiple comparisons test. CD40L (50 ng/mL), IL-21 (50 ng/mL), DMSO and zVAD-Fmk were replenished every 3 days (**A**–**H**). **P < 0.01, ***P < 0.001, ****P < 0.0001.

To explore the mechanism by which XIAP supports EBV, but not CD40L/IL-21–driven B cell outgrowth, we tested the effects of XIAP depletion on growth versus survival at early times after EBV infection. Interestingly, CRISPR *XIAP* editing significantly impaired proliferation of EBV-infected, but not CD40L/IL-21–stimulated cells (Figure 3, A and B). Furthermore, FACS analysis of 7-AAD vital dye uptake revealed an increased percentage of cell death in *XIAP*-edited and EBV-infected, but not CD40L/IL-21–stimulated, B cells (Figure 3C).

Given XIAP’s ability to block executioner caspase activity, including caspases 3 and 7 (38), we hypothesized that XIAP deficiency sensitizes EBV-infected cells to apoptosis. In support, caspase 3 and 7 activity was significantly elevated in *XIAP*-edited B cells or in XLP-2 B cells on day 4 after EBV infection, but not at the same timepoint of CD40L/IL-21 stimulation (Figure 3, D and E). We therefore tested whether caspase activity was necessary for EBV-triggered XIAP-deficient B cell apoptosis. The pan-caspase inhibitor zVAD-Fmk significantly inhibited caspase 3/7 activity and blocked *XIAP*-edited EBV-driven death (Figure 3, F and G). zVAD-Fmk also significantly increased the outgrowth of EBV-infected *XIAP* CRISPR-edited cells (Figure 3H). The pan caspase inhibitor q-VD-Oph (39) similarly rescued EBV-driven outgrowth of XIAP-deficient B cells (Supplemental Figure 3, A and B). These results further suggest that XIAP counteracts an EBV-driven apoptotic stimulus within the first week of B cell infection, a period in which EBV drives Burkitt-like hyperproliferation (12, 40, 41).

### EBV activates p53-induced apoptosis signaling.

**Figure 4:**
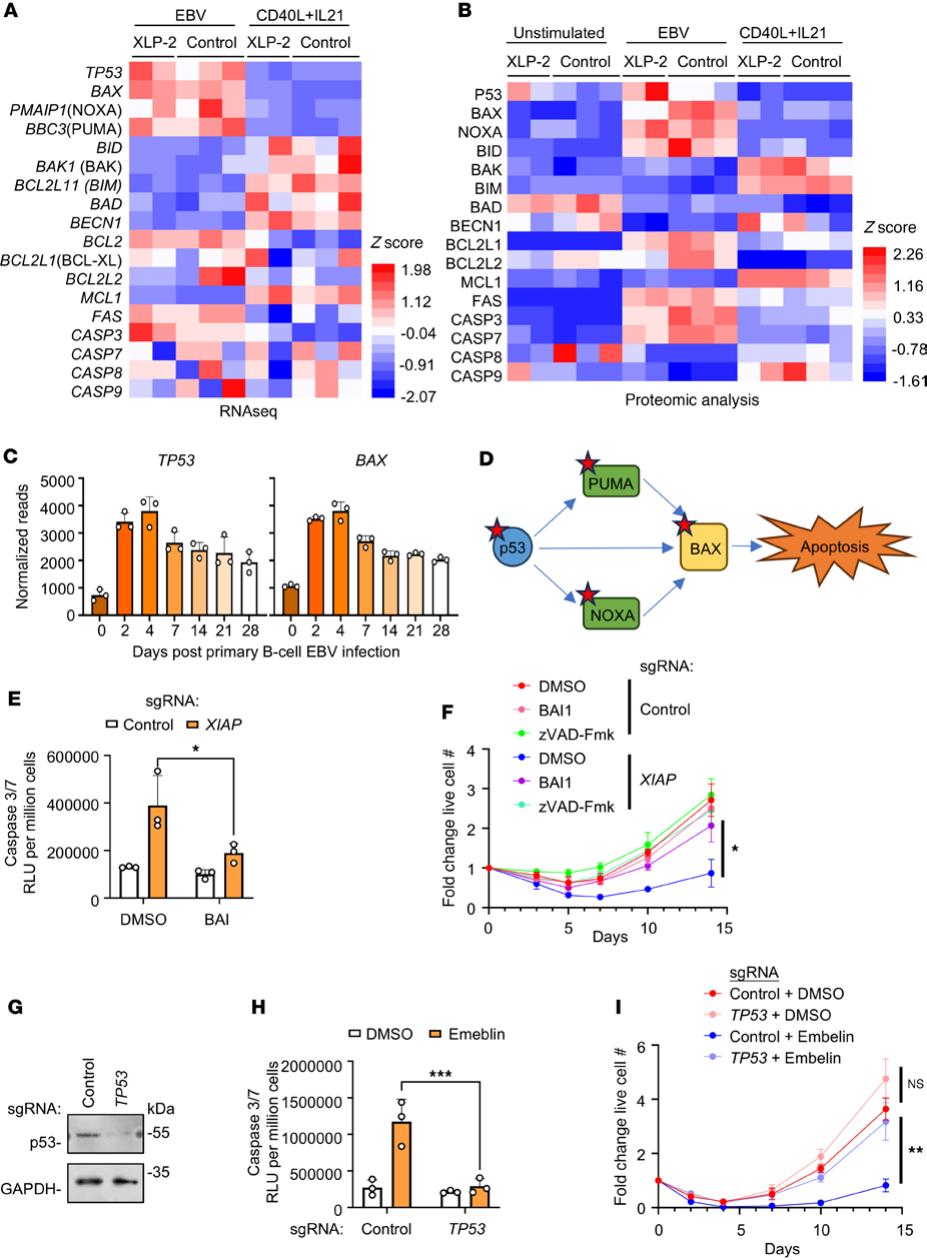
EBV but not CD40L/IL-21 activates p53- and BAX-dependent apoptosis in newly infected XIAP-deficient B cells. (**A**) RNA-seq analysis of XLP-2 patient or control primary B cells on Day 7 after EBV infection or CD40L/IL-21 treatment. Z-scores of normalized mRNA reads are shown. (**B**) Proteomic analysis of XLP-2 patient or control primary B cells on Day 7 after EBV infection or CD40L/IL-21 treatment. Unstimulated cells were harvested on Day 0. Z-scores of relative protein abundances are shown. (**C**) Mean + SD *TP53* and *BAX* mRNA levels from *n* = 3 replicates of RNA-seq of healthy donor primary B cells on the indicated days after EBV infection (43). (**D**) p53 target genes PUMA and NOXA can upregulate BAX to activate intrinsic apoptosis. Red stars denote upregulation after EBV infection relative to CD40L/IL-21 levels. (**E**) Mean + SD caspase 3/7 activity on day 4 after EBV infection from *n* = 3 replicates of primary B cells electroporated with the indicated Cas9 RNPs and treated with BAI1. (**F**) Growth curve analysis of control versus *XIAP* edited primary B cells and cultured with DMSO, zVAD-Fmk, or BAI1 from Day 0 onwards. (**G**) Immunoblot analysis of WCL from primary B cells on day 3 after electroporation with the indicated Cas9 RNPs. Blots are representative of *n* = 3 experiments. (**H**) Mean + SD caspase 3/7 activity on Day 4 after EBV infection from *n* = 3 replicates of B cells electroporated with Cas9 RNPs, EBV-infected and treated with DMSO or embelin. (**I**) Growth curve analysis of control versus *TP53* edited primary B cells cultured with DMSO or embelin from Day 0 onward. Shown are mean ± SD fold-change cell numbers, relative to uninfected values, from *n* = 3 replicates (**F** and **I**). DMSO, BAI1 (5μM), zVAD-Fmk (20 μM), and embelin (5 μM) were replenished every 3 days (**E**, **F**, **H**, and **I**). Statistical significance was assessed by 2-tailed unpaired Student’s *t* test (**F** and **I**) or 2-way ANOVA followed by Tukey’s multiple comparisons test (**E** and **H**). *P < 0.05, **P < 0.01, ***P < 0.001.

We next aimed to decipher the mechanism behind XIAP’s impact on EBV-infected but not CD40L/IL-21 proliferation. To gain insights, we performed systematic transcriptomic and whole-cell proteomic analyses of XLP-2 versus healthy control B cells at Day 7 after EBV infection or CD40L/IL-21 stimulation. This analysis highlighted that EBV upregulated expression of p53 (encoded by *TP53*) relative to levels in CD40L/IL-21–stimulated cells. EBV also altered expression levels of multiple apoptosis pathway components, increasing the proapoptotic BAX, NOXA (encoded by *PMAIP1*), and PUMA (encoded by *BBC3*), in both control and XLP-2 patient B cells (Figure 4, A and B, and Supplemental Tables 2 and 3). Notably, transient p53 upregulation within the first day of EBV B cell infection has also been described (42). Our data are generally consistent with prior transcriptomic and proteomic analyses (40, 43) of peripheral blood B cell EBV infection, which identified that p53 and BAX levels peak at day 4 after EBV infection and then gradually decline (Figure 4C).

p53, as well as multiple p53-upregulated proapoptotic proteins, can induce expression of the proapoptotic BCL-2 family member BAX (44, 45) (Figure 4D). We therefore tested whether the small molecule allosteric BAX inhibitor BAI1 (46) could suppress apoptosis induction in newly EBV-infected XIAP-deficient cells. Interestingly, BAX blockade by BAI significantly diminished EBV-driven caspase 3/7 activity in XIAP-deficient cells (Figure 4E). BAI also significantly restored EBV-mediated outgrowth of CRISPR *XIAP*-edited B cells (Figure 4F). In further support of a key p53 role, primary B cell CRISPR *TP53* KO also inhibited EBV-driven caspase 3/7 activation and rescued outgrowth of XIAP-inhibited B cells (Figure 4, G–I). Similar results were observed in CRISPR *XIAP*-depleted primary B cells treated with the small molecule p53 inhibitor pifithrin-α (47) (Supplemental Figure 4, A and B). These results suggest that p53 upregulation upon EBV infection activates BAX, which creates a dependency on XIAP to block caspase activation and apoptosis.

### XIAP deficiency heightens newly EBV-infected B cell sensitivity to IFN-γ.

**Figure 5:**
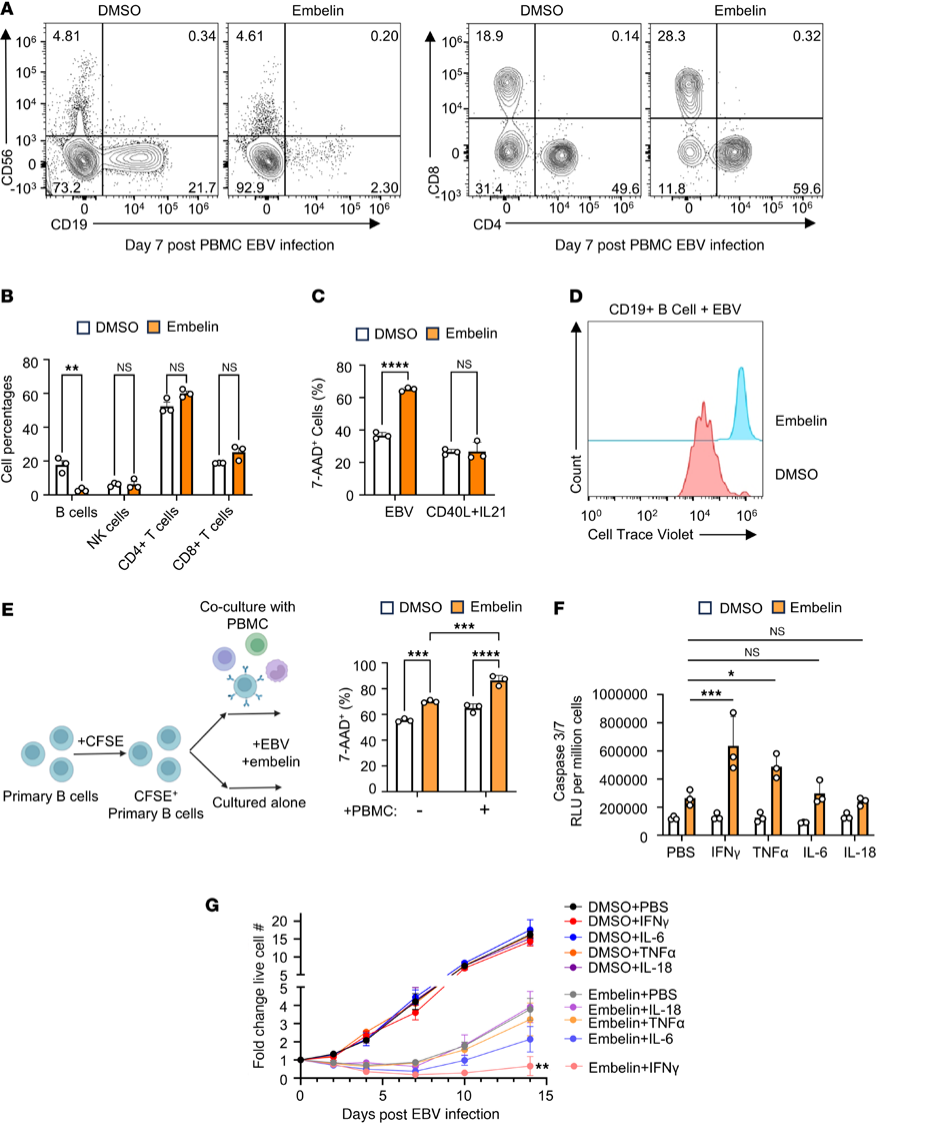
Embelin XIAP inhibition perturbs EBV-mediated primary B cell outgrowth and sensitizes newly infected cells to IFN-γ**–triggered apoptosis.** (**A**) FACS analysis of CD4^+^ or CD8^+^ T, CD56^+^ NK, and CD19^+^ B cell subsets from PBMCs of a control donor, infected with EBV and treated with DMSO or embelin, on Day 7 after EBV infection. (**B**) Mean + SD percentages of indicated cell subsets from **A** are shown. (**C**) Mean + SD %7-AAD^+^ cells from *n* = 3 replicates of DMSO or embelin-treated primary B cells on Day 4 after EBV infection or CD40L/IL21 treatment. (**D**) FACS analysis of embelin effects on infected B cell proliferation. PBMC cultures were labeled with Cell Trace Violet (CTV, whose levels are diluted by 50% with each cell division) and infected by EBV. Cells were treated with either DMSO or with embelin. CTV levels on CD19^+^ B cells from the PBMCs were measured on Day 7 after infection. (**E**) Mean + SD %7 AAD^+^ cells of primary B cells cultured alone or cocultured with autologous PBMCs and treated with either DMSO or embelin for 4 days. B cells were stained with CFSE as cell trace marker prior to PBMC coculture. (**F**) Mean + SD caspase 3/7 activity on Day 4 after infection from *n* = 3 replicates of cells treated with DMSO or embelin and also PBS, IFN-γ, TNF-α, IL-6, or IL-18. (**G**) Growth curve analysis of EBV-infected primary B cells treated with DMSO or embelin, together with IFN-γ, TNF-α, IL-6, or IL-18. Shown are mean ± SD fold-change live cell numbers from *n* = 3 replicates. Statistical significance was assessed by comparing each cytokine-treated group with PBS control group. DMSO, embelin (5 μM), and cytokines (all 50 ng/mL) were replenished every 3 days (**A**–**G**). Statistical significance was assessed by 2-tailed unpaired Student’s *t* test (**B**, **C**, and **G**) or 2-way ANOVA followed by Tukey’s multiple comparisons test (**E** and **F**). *P < 0.05, **P < 0.01, ***P < 0.001, ****P < 0.0001.

To gain insights into how XIAP deficiency alters survival of T and NK cells in EBV-infected B cell cultures, we treated human peripheral blood mononuclear cells (PBMCs) from healthy donors with embelin to inhibit XIAP. Whereas embelin significantly reduced the percentage of CD19^+^ B cells 7 days after infection, a timepoint at which most surviving B cells are EBV infected, it did not significantly reduce CD56^+^ NK, CD4^+^, or CD8^+^ T cell frequency (Figure 5, A and B). To exclude the possibility that embelin exhibits generalized B cell–specific toxicity, we treated primary B cells with embelin and then stimulated with EBV or with CD40L/IL-21. As shown in Figure 5C, Embelin selectively increased EBV^+^ B cell death. To assess proliferation, PBMC cultures were labeled with CellTrace Violet, a dye that is diluted by 50% with each round of cell division. Labelled cells were infected with EBV and treated with either DMSO or embelin, which was refreshed every 3 days. On Day 7 after infection, Cell Trace Violet levels in DMSO versus embelin-treated CD19^+^ B cells were analyzed by FACS. Strikingly, Cell Trace Levels were nearly 2 logs higher in embelin-treated than DMSO-treated cells (Figure 5D), indicating that embelin markedly blocked outgrowth of EBV^+^ cells.

While the above experiments indicate a role for XIAP in protecting newly EBV-infected cells from apoptosis, a subset of EBV^+^ B cells survive and can be immortalized, raising the question of why this does not apparently lead to lymphoma in patients with XLP-2. We therefore hypothesized that another factor may further repress EBV-mediated XIAP-deficient B cell transformation in patients with XLP-2. We noticed that embelin increased cell death of newly EBV-infected B cell death to a higher degree when they were cocultured with autologous PBMCs than when they were cultured alone (Figure 5E). This result suggested that the presence of other immune cells enhanced apoptosis of newly EBV-infected, XIAP-deficient B cells.

We hypothesized that proinflammatory cytokines might underlie this phenotype. In support, levels of IL-18, IL-6, IFN-γ, and tumor necrosis factor α (TNF-α) are elevated in XLP-2 patient serum (48). We previously found that EBV also upregulates IL-18, TNF-α, and IFN-γ mRNAs within the first week of infection (43). Since proinflammatory cytokines can sensitize cells to apoptosis, we hypothesized that XIAP deficiency and proinflammatory cytokines might exert synthetic effects to potentiate apoptosis of newly EBV-infected cells. To test this hypothesis, we treated newly EBV-infected, purified B cells from 3 healthy donors with vehicle or embelin, together with vehicle, IFN-γ, TNF-α, IL-5, or IL-18. Intriguingly, IFN-γ and to a lesser extent TNF-α, treatment significantly increased caspase 3/7 activity in embelin-treated, but not DMSO-treated EBV^+^ B cells (Figure 5F). Interestingly, IFN-γ also strongly suppressed outgrowth of embelin-treated, but not vehicle-treated B cells over the first two weeks after infection (Figure 5G). IFN-γ highly induced expression of the death receptor Fas in both control and *XIAP*-KO cells (Supplemental Figure 5), raising the possibility that Fas signaling in the infected B cell microenvironment may further restrain EBV-driven transformation in patients with XLP-2.

**Figure 6:**
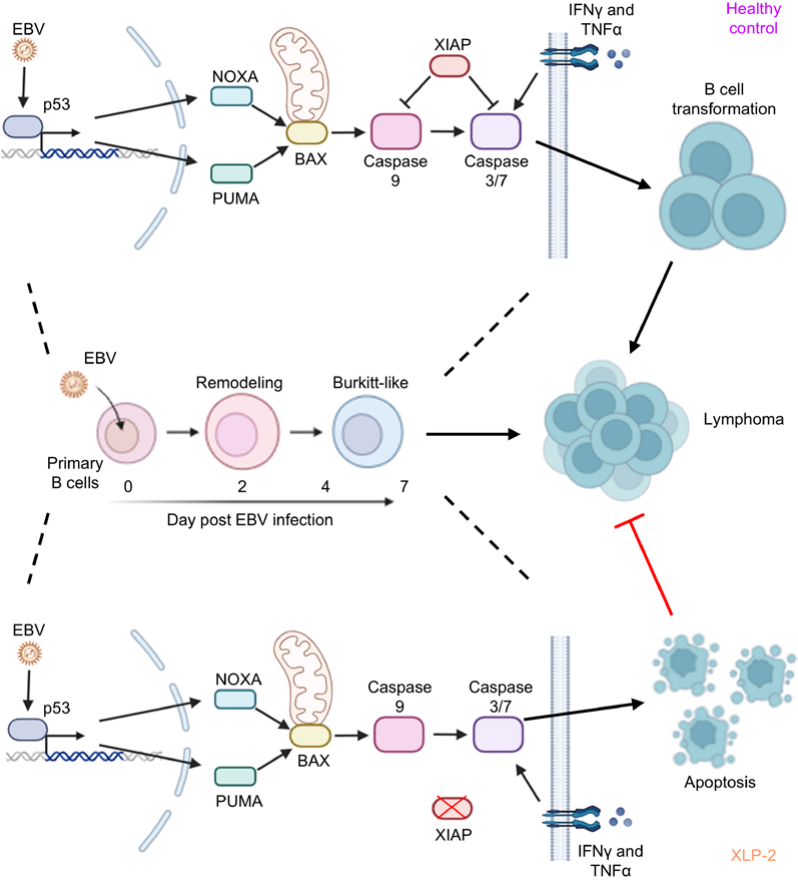
Schematic model of key antiapoptotic XIAP role in newly EBV-infected B cells. EBV drives rapid proliferation of newly infected B cells, which triggers DNA damage, upregulation of p53 and downstream NOXA, PUMA, and BAX. XIAP blocks caspase activity and apoptosis in most settings, including with XLP-1, enabling newly EBV-infected B cells to undergo transformation and in XLP-1 to cause high rates of lymphomas. Lymphomas are not observed in patients with XLP-2, where the absence of XIAP enables p53 and BAX-driven caspase 3/7 activation and apoptosis induction over the first week of EBV infection, which is exacerbated by the inflammatory cytokine milieu, in particular by IFN-γ, restraining lymphomagenesis.

Collectively, our data is consistent with a model in which EBV triggers p53-mediated apoptotic signaling during the period of Burkitt-like hyperproliferation in newly infected cells, which necessitates XIAP to inhibit downstream BAX and caspase 3/7 activation. In the absence of XIAP, EBV infection triggers p53 and BAX-dependent apoptosis, which is potentiated by inflammatory cytokines, in particular IFN-γ (Figure 6).

## Discussion

The absence of EBV-associated lymphoma is a striking feature of XLP-2, which separates it from XLP-1 and from nearly all other immunodeficiencies by the inability to control EBV infection. Why EBV-driven lymphomas are not observed in patients with XLP-2 has remained an important clinical question. Here, multi-omic profiling highlighted that in the absence of XIAP, EBV upregulates caspase 3/7 activity in a p53- and BAX-dependent manner over the first week of infection, limiting B cell transformation. Coincubation with IFN-γ or TNF-α, which are elevated in XLP-2 serum, further suppressed EBV-mediated transformation of XIAP-deficient B cells.

Our findings highlight XIAP roles in support of the earliest stages of EBV-mediated primary human B cell transformation, particularly in the presence of elevated IFN-γ or TNF-α, which are often found at elevated levels in patients with XLP-2 (48). Over the first 3 days of infection, EBV remodels B cells. EBV then drives Burkitt-like B cell hyperproliferation between days 3 and 7 after infection (12, 13, 40, 49, 50). Notably, we observed extensive cell death of *XIAP*-KO cells by day 4 after infection. EBV then progressively upregulates levels of the oncogene LMP1, which mimics aspects of CD40 signaling, to convert B cells to lymphoblastoid B cell physiology (14). Key LMP1/NF-kB pathway targets include the antiapoptotic factors cIAP1, cIAP2, cFLIP and prosurvival BCL-2 family members (18, 51). CRISPR *XIAP* KO does not significantly alter the growth or survival of established LCLs (36) and did not score in a human genome-wide CRISPR screen for LCL dependency factors (52). Taken together, these results suggest that XIAP exerts key prosurvival roles in the early EBV-driven hyperproliferation stage of newly infected B cells, where markedly elevated levels of EBNA2 and MYC are observed, prior to the upregulation of antiapoptotic factors by LMP1 and perhaps also prior to maximal inhibition of p53 by EBNA3C (53, 54).

What then triggers apoptosis signaling within the first week of EBV infection? EBV-driven B cell hyperproliferation activates DNA damage responses (DDR), which signal through p53 (12, 50, 55, 56). Following the initial phase of rapid proliferation, by approximately day 7 after EBV infection, the proliferation rate slows. Our results suggest that such elevated p53 levels, and upregulation of p53 targets including NOXA and PUMA, activate BAX, which is a 3 Bcl2 homology domain 3–only (BH3-only) apoptosis effector. When expressed at elevated levels and not counteracted by prosurvival BCL2 family members, BAX undergoes conformational changes, oligomerization, and insertion into the mitochondrial outer membrane. This releases cytochrome c and other apoptogenic factors to activate executioner caspase activity, including caspases 3 and 7 (44, 45). Thus, our results suggest that EBV-driven B cell hyperproliferation and DNA damage signaling creates a dependency on XIAP prior to high levels of LMP1 expression, particularly in a hyperinflammatory cytokine milieu. This may also explain why XIAP is not required for B cell proliferation driven by CD40L/IL-21, as CD40 signaling overlaps substantially with that of LMP1 and upregulates antiapoptotic factors, including cIAP1 and 2.

IFN-γ–producing NK cells can block EBV-mediated B cell transformation (57), and we found that XIAP-deficient B cells are exquisitely sensitive to IFN-γ following EBV infection. p53 and IFN-γ have an intricate relationship, and it is plausible that IFN-γ heightens sensitivity to p53-driven apoptosis (58) in newly EBV-infected B cells. For instance, IFN-γ can upregulate nuclear p53 levels and enhance its interaction with target genes (59). In addition, IFN-γ regulates a broad range of apoptosis-related molecules, including several p53 transcriptional targets. According to the Interferome database (60), IFN-γ significantly upregulates BAK1, PUMA (BBC3), and NOXA (PMAIP1), all of which contribute to mitochondrial apoptotic priming. IFN-γ also enhances expression of ROS-generating enzymes such as NOX1 and DUOX2, which can produce oxidative stress that may further sensitize newly infected cells to apoptosis (61, 62). Alternatively, XLP-2 lymphocytes exhibit increased susceptibility to extrinsic apoptosis triggered by Fas, TNF-α, or TNF-related apoptosis-inducing ligand (TRAIL) (28, 63). We found that IFN-γ markedly upregulated Fas in WT and XIAP-deficient B cells. Although FasL does not appear to be expressed on EBV^+^ B cells, when highly expressed, Fas can initiate apoptotic signaling, even in the absence of FasL, through ligand-independent oligomerization and clustering into membrane microdomains termed Fas caps. This promotes local receptor concentration, and recruitment of downstream FADD and caspase-8, thereby triggering apoptosis (64–66). Since EBV also upregulates Fas, further IFN-γ–driven Fas upregulation may therefore sensitize newly infected cells to apoptosis in the absence of XIAP. Notably, p53 binds to multiple *FAS* gene promoter and intronic elements and upregulates its transcription (67–69). TNF-α may similarly trigger apoptosis signaling in newly EBV-infected B cells in the absence of XIAP, as is observed in *XIAP*-KO murine bone marrow–derived macrophages and in *XIAP*-KO mice in vivo (63). We note that EBV^+^ lymphoblastoid cells are also dependent on cFLIP to block TNF-α–mediated apoptosis (52).

In addition to apoptosis, other forms of programmed cell death may contribute to the increased susceptibility of XIAP-deficient B cells following EBV infection. However, we note that caspase or BAX inhibition rescued proliferation of EBV-infected XIAP-deficient cells, suggesting that apoptosis is major programmed cell death pathway activated at early timepoints of EBV infection in the absence of XIAP. While we recently demonstrated that newly EBV-infected B cells are particularly susceptible to ferroptosis (70), we are not aware of evidence that XIAP can alter lipid metabolism, generation of lipid ROS, or detoxification of ROS. Nonetheless, IAP protein inhibition, combined with ferroptosis-inducing agents, can synergistically enhance lipid peroxidation and cell death in acute lymphoblastic leukemia cells (71). This observation raises the possibility that XIAP deficiency may lower the threshold for ferroptosis induction in newly EBV-infected cells. XIAP has also been implicated in the regulation of necroptosis and autophagy (72, 73), suggesting that disruption of XIAP function may facilitate multiple death signaling pathways. The involvement of these cell death pathways remains a possibility and merits further investigation.

LCLs derived from patients with XLP-2 have modestly elevated levels of EBV lytic gene expression (36). However, we did not observe significantly elevated lytic gene expression in our transcriptomic or proteomic profiling, suggesting that this phenomenon may be specific to established LCLs. The tumor suppressor cell adhesion molecule 1 (CADM1) was also highly upregulated on LCLs established from XLP-2 B cells (36). Consistent with this report, our proteomic profiling identified CADM1 upregulation in XLP-2 B cells 7 days after infection (Supplemental Table 2), suggesting that this is an early phenomenon in EBV-infected XLP-2 B cells. CADM1 is also highly upregulated by Kaposi’s Sarcoma Associated Herpesvirus B cell infection, potentially suggesting an oncogenic role (74). While CADM1 KO did not impair LCL proliferation (36), it will be of interest to define effects of CADM1 KO on newly EBV-infected control versus XIAP-deficient B cells.

In summary, we identified a key role for XIAP in blockade of EBV-induced apoptosis signaling within the first week of infection. Loss of XIAP function impaired proliferation and triggered apoptosis of EBV^+^ B cells, particularly at the Burkitt-like hyperproliferation stage and prior to full LMP1 upregulation and prosurvival signaling. This proapoptotic pathway was dependent on p53 and BAX and was exacerbated by IFN-γ or TNF-α, the levels of which are elevated in XLP-2 patient serum. Our results provide insights into the curious absence of EBV-driven lymphoproliferative disease in patients with XLP-2, despite heightened sensitivity to EBV.

## Methods

### Sex as a biological variable.

Since XLP-2 is an X-linked recessive disease, it typically occurs only in males, and all of the XLP-2 patients mentioned in the study are male. The primary human B cells used in the study came from deidentified donors, which likely included both males and females.

### Cell cultures.

HEK293T cells were purchased from ATCC and cultured in DMEM medium containing 10% FBS. GM12878 and GM15892 Cas9^+^ lymphoblastoid cell lines were purchased from Coriell and maintained in RPMI-1640 supplemented with 10% FBS and 5 μg/mL blasticidin. All cells were incubated in a humidified incubator at 37°C with 5% CO_2_ and were routinely confirmed to be mycoplasma negative using the MycoAlert kit (Lonza).

### Chemical compounds.

Proliferation of primary B cells was induced by 50 ng/mL rhCD40L (Enzo Life Sciences) plus 50 ng/mL rhIL-21 (Biolegend). For the inhibition of XIAP, 5 μM embelin (Selleck) was used. 20 μM zVAD-Fmk (Selleck) and 10 μM q-VD-Oph (Selleck) were used to inhibit caspase 3 and 7. 5 μM BAI1 (MedChemexpress) and 5 or 20 μM Pifithrin-α (MedChemexpress) were used for inhibition of Bax and p53, respectively. All reagents were replenished every 3 days.

### Antibodies.

Anti-XIAP (Cell Signaling Technology, 3B6, #2045), cIAP1 (Proteintech, 1H3F1, #66626-1-IG), cIAP2 (Cell Signaling Technology, 58C7, #3130), EBNA1 (mouse monoclonal antibody OT1X, a gift from Jaap Middeldorp), p53 (Cell Signaling Technology, #9282), DDX1 (Bethyl Laboratories, #A300-521A), and GAPDH (EMD Millipore, #MAB374) antibodies were used for immunoblot assay. Murine monoclonal antibody S12 against LMP1 (75) was purified from hybridoma (generously provided by Elliott Kieff) supernatant and used at 1:1,000 for immunoblot analysis. BioLegend antibodies against CD95/FAS (DX2, #305612), CD4 (OKT4, #317416), CD8 (RPA-T8, #301006), CD56 (5.1H11, #362503), and CD19 (4G7, #392507) were used for flow cytometry analysis.

### Primary human B cells.

Leukocyte fractions that were discarded and deidentified, originating from platelet donations, were obtained from the Brigham and Women’s Hospital Blood Bank. These fractions were utilized for the isolation of primary human B cells following our IRB-approved protocol. Venous blood of patients with XLP-2 and corresponding individuals in a control group were obtained from Boston Children’s Hospital. PBMCs were isolated using Lymphopre Density Gradient Medium (Stem Cell Technologies), and primary B cells were subsequently isolated by negative selection using RosetteSep Human B Cell Enrichment and EasySep Human B cell enrichment kits (Stem Cell Technologies), according to the manufacturer’s protocols. Cells were cultured in RPMI-1640 medium with 10% FBS.

### EBV production.

For production of Akata EBV, Akata EBV^+^ cells were resuspended in FBS-free RPMI-1640 at a concentration of 2–3 × 10^6^ cells per mL and treated by 0.3% Polyclonal Rabbit Anti-Human IgG (Agilent) for 6 hours. Cells were cultured in RPMI-1640 with 4% FBS for 3 more days, and the virus-containing supernatants were collected by ultracentrifugation and filtration through a 0.45 μm filter. The viral titer was determined by EBV transformation assay, as described below.

### EBV transformation assay.

Purified human primary B cells were seeded into a 96 well plate at 50,000 cells per well. The stock of Akata EBV was serially diluted 10-fold, in order, and 100 μL of virus dilution was added to each well. The cells were maintained in RPMI-1640 with 10% FBS at 37°C. After 4 weeks of incubation, the proportion of wells with B cell outgrowth was scored. A transforming unit per well was defined as the virus quantity necessary for achieving B cell outgrowth in 50% of wells. The multiple of infection (MOI) was determined by dividing the Transforming Unit by the cell number.

### CRISPR/Cas9 editing.

For cell lines with stable Cas9 expression, sgRNA sequences from Broad Institute Brunello library were used. sgRNA oligos were cloned into the pLentiGuide-Puro vector (Addgene plasmid #52963, a gift from Feng Zhang (Broad Institute of MIT and Harvard, Cambridge, Massachusetts, USA)), and used for lentivirus production in HEK293T cells. After 2 rounds of transduction performed at 48 and 72 hours after plasmid transfection, cells were selected by 3 μg/mL puromycin for more than 4 days.

For CRISPR/Cas9 editing in primary B cells, Cas9 RNA complexes were electroporated into 1 million cells. In brief, CRISPR RNA (crRNA) targeting *XIAP* or *TP53* was selected using Alt-R Predesigned Cas9 crRNA Selection Tool from Integrated DNA Technologies. Nontargeting control crRNA from Integrated DNA Technologies was used as a control for the electroporation. Trans-activating CRISPR RNA (tracrRNA) and Cas9 Nuclease V3 were also obtained from Integrated DNA Technologies. The crRNA and tracrRNA were annealed to form the duplex and incubated with Cas9 for 20 minutes. Then the cells were mixed with the RNP complexes and electroporated using the Neon NxT Electroporation System at 1,700V, 20ms, and 2 pulses. sgRNA sequences are listed in Supplemental Table 1.

### Immunoblot analysis.

Cells were lysed in 1× Laemmli buffer with ultrasound, and then boiled at 95°C for 10 minutes. The proteins were separated by SDS-PAGE electrophoresis and transferred onto the nitrocellulose membranes. The membrane was blocked with 5% milk in TBST buffer and incubated with primary antibodies at 4°C overnight, followed by 1 hour incubation with secondary antibodies at room temperature. Blots were then developed by incubation with ECL chemiluminescence for 1 min (Millipore) and images were captured by Licor Fc platform.

### Flow cytometry analysis.

For live cells staining, 1 × 10^6^ of cells were washed twice with FACS buffer (PBS containing 1% FBS), followed by primary antibody incubation at dark for 30 minutes. For 7-AAD staining, cells were stained with 7-AAD for 10 minutes on ice. Then the stained cells were washed with FACS buffer and subjected to flow cytometry analysis. Data was analyzed with FlowJo X software (FlowJo).

### Proliferation assay.

Cells were seeded in a 12 well plate at 0.5 million cells per well and treated with indicated reagents. Culture medium was changed every 3 or 4 days, and the live cell numbers were counted on indicated days using trypan blue staining. Cell counts relative to day 0 were calculated and used for the plots.

### Carboxyfluorescein succinimidyl ester and cell trace violet staining.

For Carboxyfluorescein succinimidyl ester *(*CFSE) or cell trace violet (CTV) staining, cells were washed twice with PBS, followed by staining with 1 μM CFSE or CTV in the dark for 20 minutes. Then the cells were washed twice with RPMI-1640 containing 10% FBS and subjected to subsequent experiments.

### Caspase-Glo 3/7 assay.

Caspase 3/7 activity was assessed using Caspase-Glo 3/7 Assay (Promega) according to manufacturer’s instructions. In brief, cells were mixed with Caspase-Glo 3/7 reagent at a ratio of 1:1 and incubated for 1 hour at room temperature. The luminescence signal was assessed with a Molecular Devices microplate reader. The final presentation of caspase-3/7 activities was established by normalizing the signal against the count of viable cells, determined through trypan blue staining.

### RNA-seq analysis.

Total RNA was extracted from the cells using RNeasy Mini kit (Qiagen), following the manufacturer’s instruction. A DNA digestion step within the column was incorporated to eliminate any remaining genomic DNA contamination. For library construction, poly(A) mRNA was isolated from 1 μg DNA-free total RNA using NEBNext Poly(A) mRNA Magnetic Isolation Module (New England Biolabs), followed by library construction via the NEBNext Ultra RNA Library Prep Kit (New England Biolabs). Library quality was assessed by an Agilent Bioanalyzer DNA Chip. Multiindexed libraries were combined, pooled, and subjected to sequencing on an Illumina NextSeq 500 sequencer with single-end 75 bp reads at the Dana Farber Molecular Biology core. Raw read counts for gene expression were quantified using salmon v1.2.0 with human GENCODE v28 (GRCh37) genes. Differential expressions were evaluated by DESeq2.

### Mass spectrometry analysis.

Cells were lysed in (50 mM Tris-HCI pH 7.5, 300 mM NaCl, 0.5% v/v NP40, 1 mM DTT and Roche protease inhibitor cocktail). Proteins were precipitated with 20% trichloroacetic acid (TCA), washed once with 10% TCA, washed 3 times with cold acetone, and dried to completion, using a centrifugal evaporator. Samples were resuspended in digestion buffer (50 mM Tris-HCl pH 8.5), 10% acetonitrile (AcN), 1 Mm DTT, and 10 mg/mL trypsin (Promega), then incubated overnight at 37°C with agitation. The reaction was quenched by 50% formic acid (FA), subjected to C18 solid-phase extraction and vacuum-centrifuged to complete dryness. Samples were reconstituted in 4% AcN/5% FA and divided into technical duplicates prior to LC-MS/MS on an Orbitrap Lumos.

### Statistics.

Unless otherwise indicated, all bar graphs and line graphs represent the arithmetic mean of 3 independent experiments (*n* = 3), with error bars denoting SDs. The statistical significance between different groups was assessed using the unpaired Student’s *t* test or ANOVA) with the appropriate post test using GraphPad Prism 9 software. *P* less than 0.05 was considered significant.

### Study approval.

Our studies on primary human blood cells were approved by the Brigham & Women’s Hospital Institutional Review Board under 2004p002711 and Boston Children’s Hospital Institutional Review Board under 04-09-113R. For patients with XLP-2, written informed consent was received prior to participation.

### Data availability.

RNA-seq data was deposited in the SRA database under BioProject accession number PRJNA1259017. Original data values are provided in the Supporting Data Values File. Requests for resources and reagents can be directed to the first author or corresponding author. Figures were drawn with GraphPad, Biorender and Microsoft Powerpoint.

## Author contributions

YS performed the experiments, data analysis, wrote the first draft, and edited the manuscript together with BEG. JC provided blood samples of XLP-2 patients and healthy controls. KDD and SPG performed mass spectrometry analysis. BEG supervised the study. All authors read and approved the final manuscript.

## Supplementary Material

Supplemental data

Unedited blot and gel images

Supplemental table 2

Supplemental table 3

Supporting data values

## Version 1 07/17/2025

In-Press Preview

## Version 2 08/22/2025

Electronic publication
