# Supplementary material for: Insights into absence of lymphoma despite fulminant Epstein-Barr virus infection in patients with XIAP deficiency
Source: JCI Insight. 2025 Jul 15;10(16):e193787. doi: 10.1172/jci.insight.193787 (PMC12406724; doi:10.1172/jci.insight.193787)
Supplement: Supplemental data [file jciinsight-10-193787-s242.pdf]

Supplementary Figure legends and tables

Figure S1

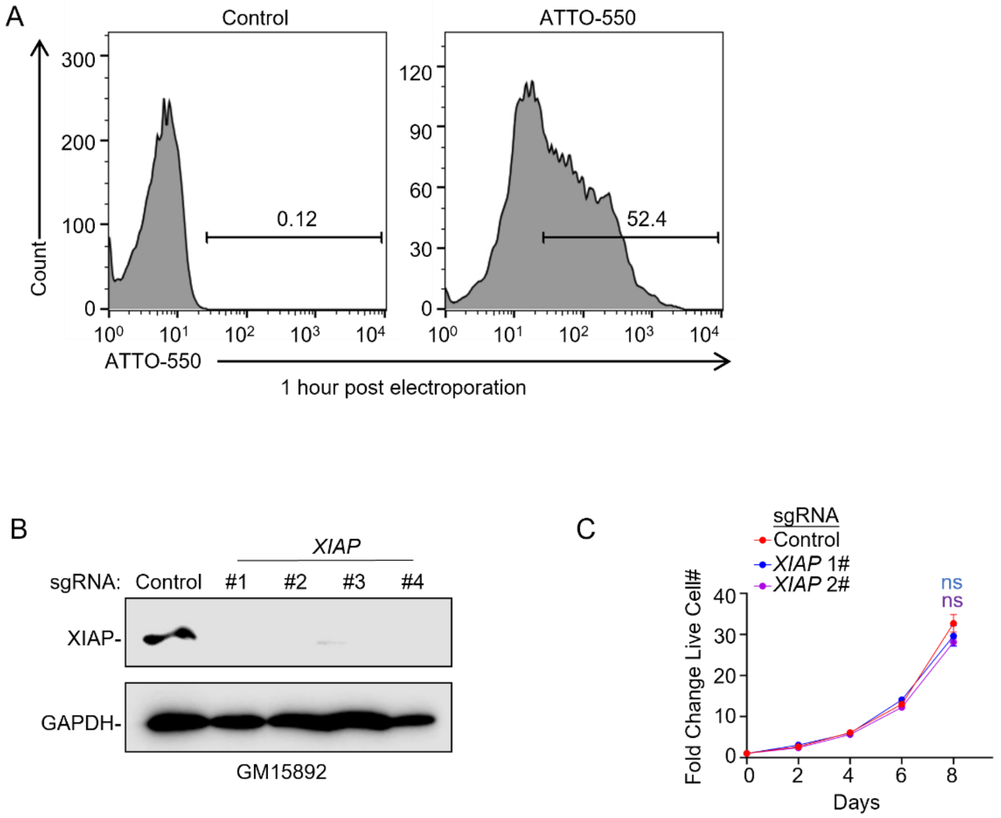

Figure S1. CRISPR *XIAP* KO does not alter the growth or survival of GM15829 LCLs.

(A) FACS analysis of primary human B cells electroporation efficiency. Shown are FACS plots of primary human B-cells at 1-hour post-electroporation with control unlabeled versus ATTO™ 550 conjugated tracrRNA duplex.

(B) Immunoblot analysis of WCL from Cas9+ GM15892 LCLs that expressed the indicated control or one of four independent *XIAP*-targeting sgRNAs.

(C) Growth curve analysis of Cas9+ GM15892 cells expressing control or *XIAP* targeting sgRNAs. Shown are mean ± SD fold change live cell numbers from n=3 replicates, relative to Day 0 values.

Statistical significance was assessed by one-way ANOVA followed by Tukey's multiple comparisons test (C). Blots are representative of n=3 replicates. ns, not significant.

Figure S2

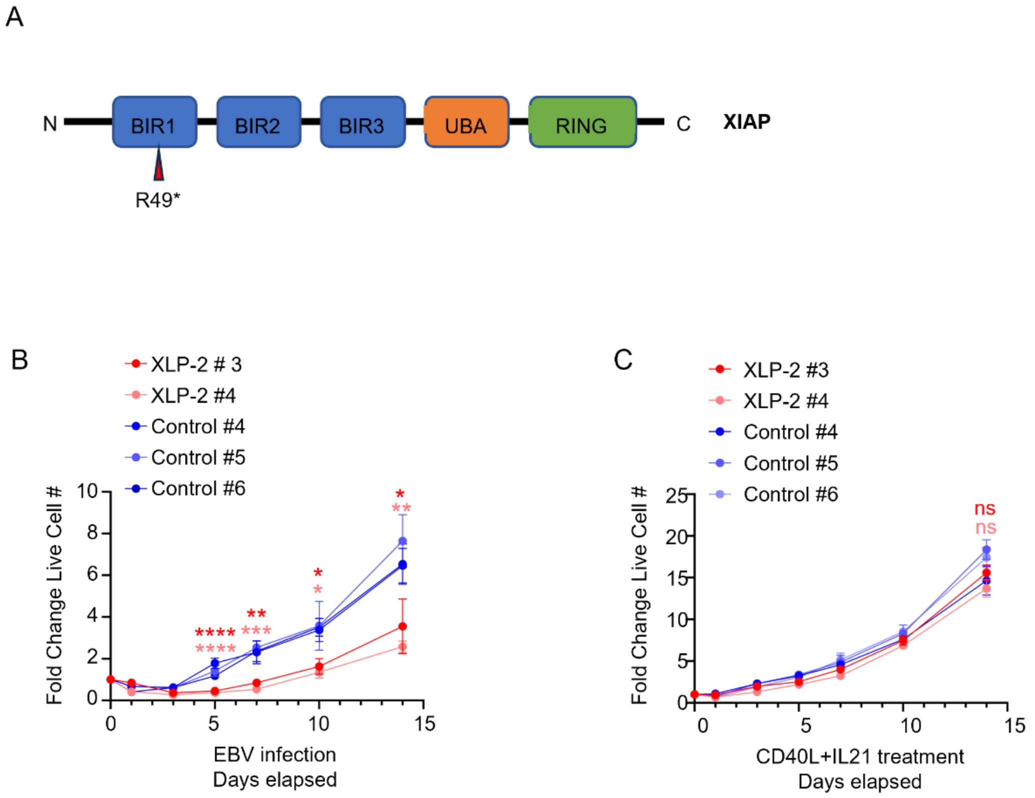

Figure S2. Outgrowth of XLP-2 cells is impaired at early time points of EBV infection but not of CD40L/IL-21 treatment.

(A) Schematic diagram highlighting the *XIAP* mutation shared by XLP-2 Patients #3 and #4.

(B) Growth curve analysis of primary B-cells from XLP-2 patients versus controls following EBV infection. Shown are mean ± SD fold change live cell numbers from n=3 replicates of B-cells infected at Day 0 with EBV, relative to Day 0 values. The annotations represent the results of statistical comparisons between XLP-2 samples and Control #4.

(C) Growth curve analysis of primary B-cells from XLP-2 patients versus controls stimulated by CD40L/IL-21. Shown are mean ± SD fold change live cell numbers from n=3 replicates of B-cells stimulated by CD40L and IL-21, which were replenished every 3 days. The annotations represent the results of statistical comparisons between XLP-2 samples and Control #4.

Statistical significance was assessed by one-way ANOVA followed by Tukey's multiple comparisons test (B and C). *, $p<0.05$; **, $p<0.01$; ***, $p<0.001$; ****, $p<0.0001$; ns, not significant.

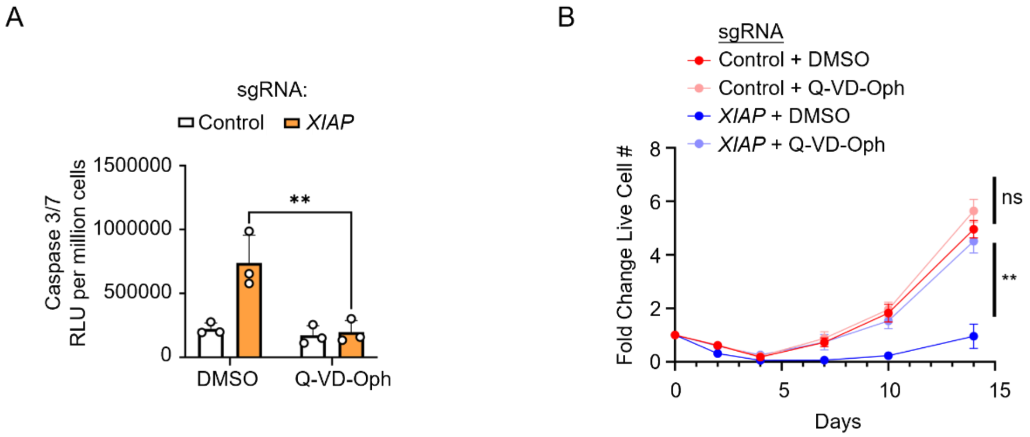

Figure S3. EBV triggers caspase activation and apoptosis in XIAP deficient B-cells.

(A) Mean + SD caspase 3/7 activity on Day 4 post-EBV infection from n=3 replicates of control or *XIAP* edited primary B-cells. Primary cells were electroporated on Day 0 with Cas9 RNPs loaded with control or *XIAP* targeting sgRNAs and EBV-infected. Cells were grown in the presence of DMSO vehicle or the pan-caspase inhibitor Q-VD-Oph (10 μM). DMSO or Q-VD-Oph were added on Day 0 and refreshed on Day 3.

(B) Growth curve analysis of control versus *XIAP* edited primary human B-cells infected with EBV on Day 0 and cultured with DMSO vehicle or Q-VD-Oph (10 μM). Shown are mean ± SD fold change live cell numbers, relative to uninfected values from n=3 replicates. DMSO or Q-VD-Oph were added at Day 0 and replenished every 3 days.

Statistical significance was assessed by two-way ANOVA followed by Tukey's multiple comparisons test (A) or two-tailed unpaired Student's t test (B). **, p<0.01; ns, not significant.

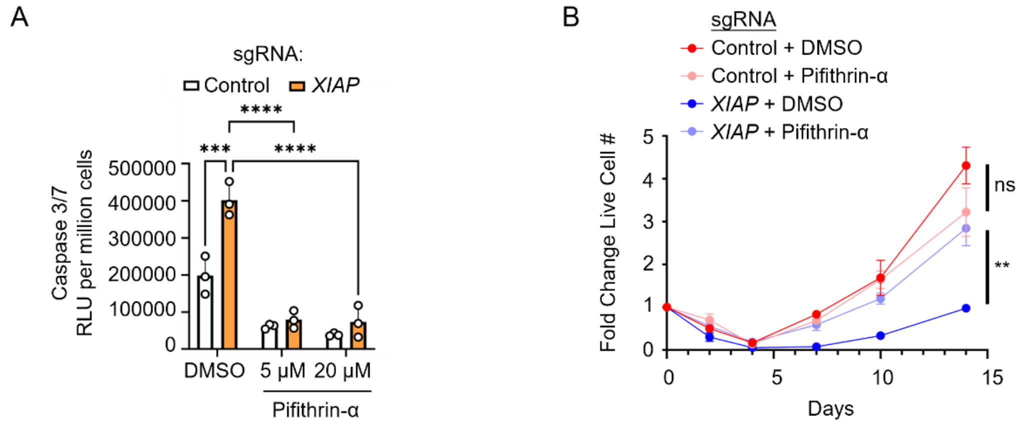

Figure S4. p53 signaling is required for the apoptosis of EBV-infected, XIAP deficient B-cells.

(A) Mean + SD caspase 3/7 activity on Day 4 post-infection from n=3 replicates of control versus *XIAP* edited primary B-cells cultured with DMSO or pifithrin-α at 5 or 20 µM. DMSO or pifithrin-α were added on Day 0 and refreshed on Day 3.

(B) Growth curve analysis of control versus *XIAP* edited primary B-cells infected with EBV on Day 0 and cultured with DMSO vehicle or pifithrin-α (5 µM). Shown are mean ± SD fold change live cell numbers from n=3 replicates, relative to uninfected values. DMSO or pifithrin-α were added at Day 0 and replenished every 3 days.

Statistical significance was assessed by two-way ANOVA followed by Tukey's multiple comparisons test (A) or two-tailed unpaired Student's t test (B). **, $p<0.01$; ***, $p<0.001$; ****, $p<0.0001$; ns, not significant.

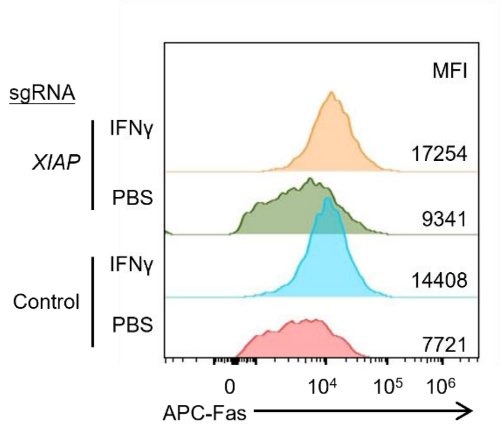

Figure S5. IFNγ induces Fas in control and XIAP deficient EBV-infected B-cells.

FACS analysis of plasma membrane Fas expression on control versus *XIAP* edited primary B-cells at Day 4 post-EBV infection. Cells were infected on Day 0 and cultured with PBS vehicle or IFNγ (50 ng/mL), which was replenished on Day 3. MFI, mean fluorescence index. Plots are representative of n=3 replicates.

Table S1. sgRNA sequence used in this study

sgRNA name	sgRNA sequence
Control	ATTTCGCAGATCATCGACAT
TP53 #1 (for primary B cells)	CCATTGTTCAATATCGTCCG
TP53 #2 (for primary B cells)	TCCACTCGGATAAGATGCTG
XIAP #1 (for primary B cells)	GCATCAACACTGGCACGAGC
XIAP #2 (for primary B cells)	AGTGCTGGACTCTACTACAC
XIAP #1 (for cell lines)	ATGACAACTAAAGCACCGCA
XIAP #2 (for cell lines)	ATGGATATACTCAGTTAACA
XIAP #3 (for cell lines)	TCTGACCAGGCACGATCACA
XIAP #4 (for cell lines)	TATCAGACACCATATACCCG
