# Supplementary material for: Insights into absence of lymphoma despite fulminant Epstein-Barr virus infection in patients with XIAP deficiency
Source: JCI Insight. 2025 Jul 15;10(16):e193787. doi: 10.1172/jci.insight.193787 (PMC12406724; doi:10.1172/jci.insight.193787)

Figure 1B

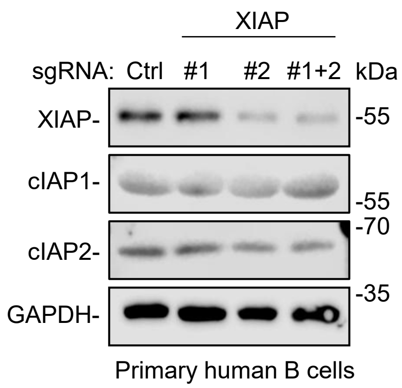

For these blots, the same batch of samples was run at the same time in parallel on separate gels under identical conditions

Anti-XIAP

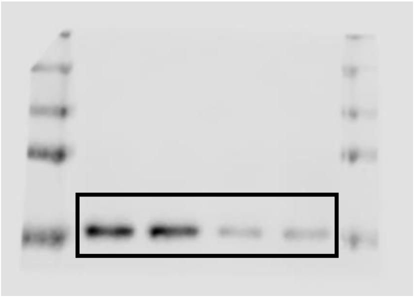

Anti-GAPDH

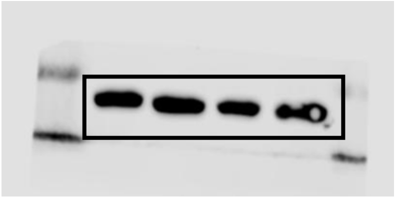

Anti-cIAP1

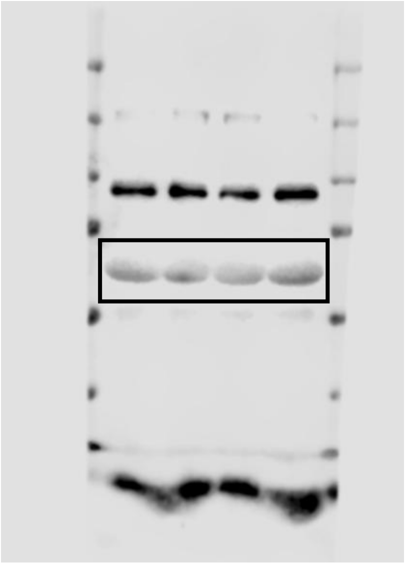

Anti-cIAP2

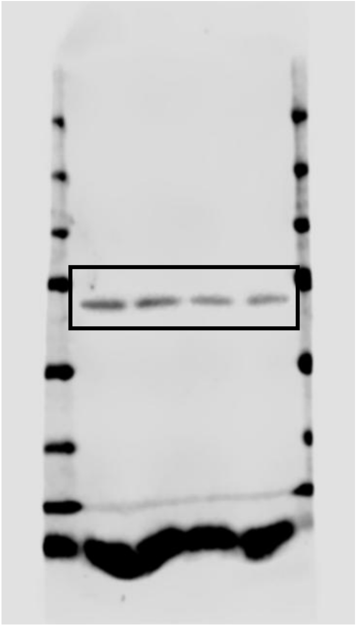

Figure 1E

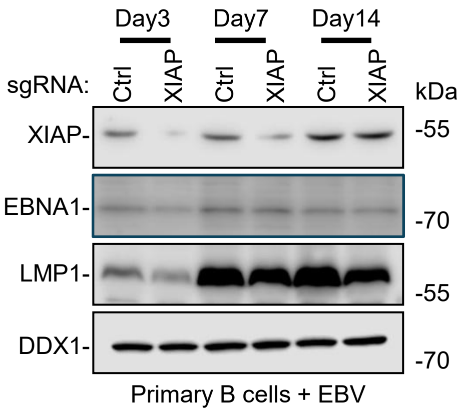

For these blots, the same batch of samples was run at the same time in parallel on separate gels under identical conditions

Anti-XIAP

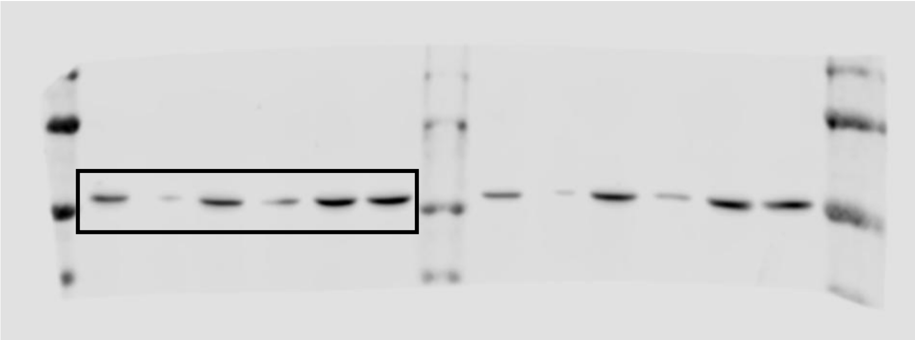

Anti-EBNA1

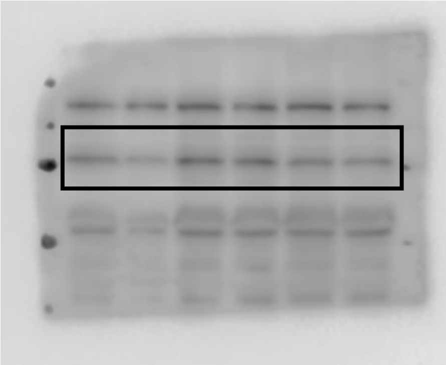

Anti-LMP1

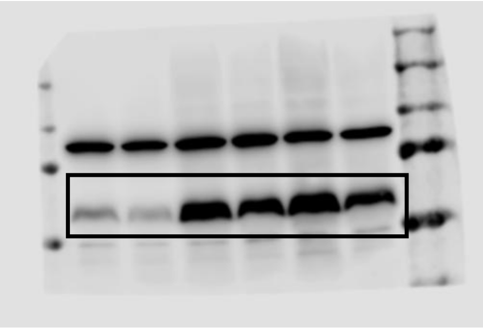

Anti-DDX1

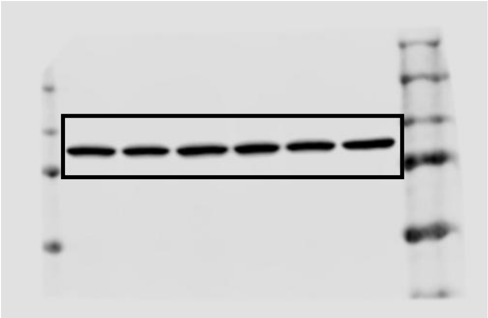

Figure 1G

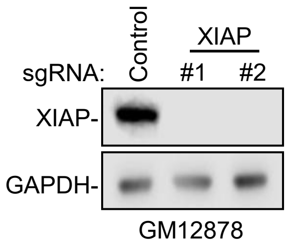

Anti-XIAP

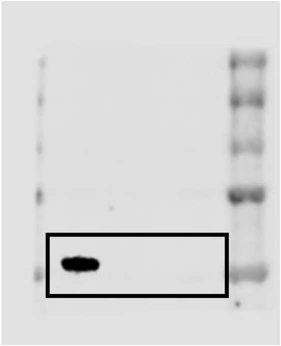

Anti-GAPDH

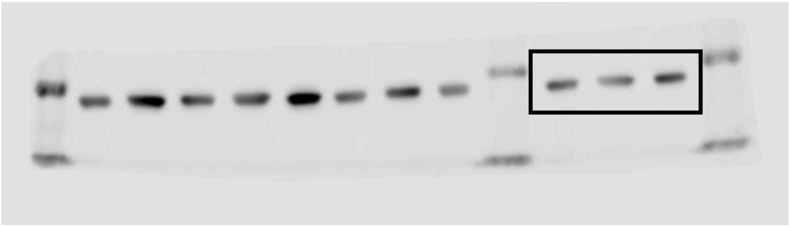

For Figure 1G, the XIAP and GAPDH blots were obtained from the same gel. After electrophoresis and transfer, the same membrane was cut twice. First, we separated top and bottom sections. The bottom section was probed for GAPDH load control. The top piece was cut once more, within the lane 10 protein ladder. This separated Lanes 1-9, which were used for a separate experiment, from lanes 11-14, which were probed for anti-XIAP and presented in Figure 1G.

Figure S1B

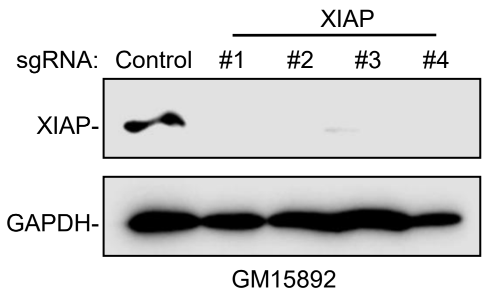

Anti-XIAP

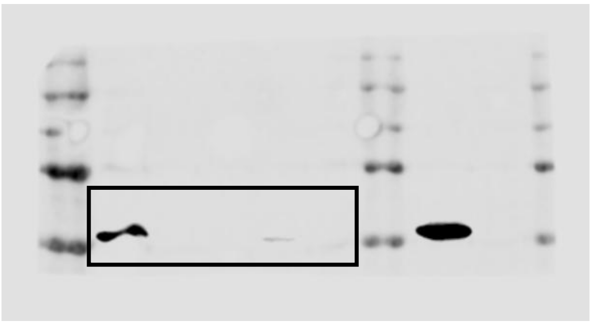

Anti-GAPDH

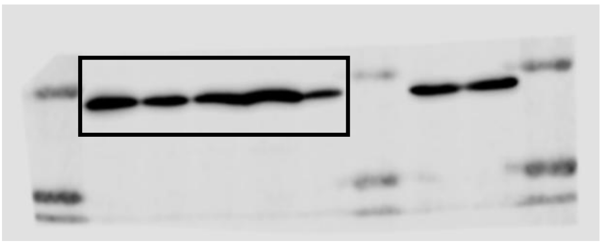

Figure 4G

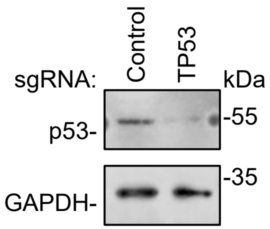

Anti-p53

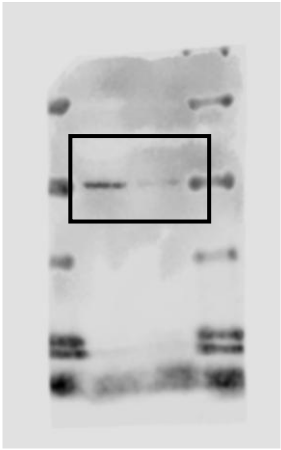

Anti-GAPDH

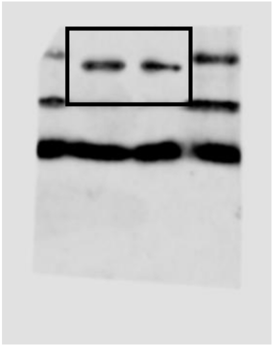

Supplement: Unedited blot and gel images [file jciinsight-10-193787-s243.pdf]
